# Phosphatidylserine-mediated platelet clearance by endothelium decreases platelet aggregates and procoagulant activity in sepsis

**DOI:** 10.1038/s41598-017-04773-8

**Published:** 2017-07-10

**Authors:** Ruishuang Ma, Rui Xie, Chengyuan Yu, Yu Si, Xiaoming Wu, Lu Zhao, Zhipeng Yao, Shaohong Fang, He Chen, Valerie Novakovic, Chunyan Gao, Junjie Kou, Yayan Bi, Hemant S. Thatte, Bo Yu, Shufen Yang, Jin Zhou, Jialan Shi

**Affiliations:** 1 Department of Hematology of the First Hospital, Harbin Medical University, Harbin, China; 2 The Key Laboratory of Myocardial Ischemia, Ministry of Education, Heilongjiang Province, Harbin Medical University, Harbin, China; 3 Department of Medicine of the Third Hospital, Harbin Medical University, Harbin, China; 4 Department of Cardiology of the Second Hospital, Harbin Medical University, Harbin, China; 5 Departments of Cardiology of the First Hospital, Harbin Medical University, Harbin, China; 6 Department of Pathology, Harbin Medical University, Harbin, China; 7 Departments of Research VA Boston Healthcare System, Harvard Medical School, Boston, Massachusetts USA; 8 Departments of Surgery, Brigham and Women’s Hospital, VA Boston Healthcare System, Harvard Medical School, Boston, Massachusetts USA

## Abstract

The mechanisms that eliminate activated platelets in inflammation-induced disseminated intravascular coagulation (DIC) in micro-capillary circulation are poorly understood. This study explored an alternate pathway for platelet disposal mediated by endothelial cells (ECs) through phosphatidylserine (PS) and examined the effect of platelet clearance on procoagulant activity (PCA) in sepsis. Platelets in septic patients demonstrated increased levels of surface activation markers and apoptotic vesicle formation, and also formed aggregates with leukocytes. Activated platelets adhered were and ultimately digested by ECs *in vivo* and *in vitro*. Blocking PS on platelets or αvβ3 integrin on ECs attenuated platelet clearance resulting in increased platelet count in a mouse model of sepsis. Furthermore, platelet removal by ECs resulted in a corresponding decrease in platelet-leukocyte complex formation and markedly reduced generation of factor Xa and thrombin on platelets. Pretreatment with lactadherin significantly increased phagocytosis of platelets by approximately 2-fold, diminished PCA by 70%, prolonged coagulation time, and attenuated fibrin formation by 50%. Our results suggest that PS-mediated clearance of activated platelets by the endothelium results in an anti-inflammatory, anticoagulant, and antithrombotic effect that contribute to maintaining platelet homeostasis during acute inflammation. These results suggest a new therapeutic target for impeding the development of DIC.

## Introduction

Disorders of coagulation are common in sepsis, with disseminated intravascular coagulation (DIC) occurring in approximately 35% of severe cases, contributing to microvascular dysfunction and death^1,2^. Intensive platelet activation in sepsis facilitates platelet aggregation, leading to the formation of microthrombi and platelet depletion^3^ with the resultant development of DIC and sepsis-associated thrombocytopenia. Therefore, platelets must be cleared locally and in a timely fashion in the early phase of activation. Previous studies mainly focused on the clearance of activated cold-stored and aging platelets as well as platelets in immune-mediated thrombocytopenia^4,5^, however, platelet activation and their clearance in sepsis are poorly understood. Platelets can form aggregates with leukocytes resulting in leukocyte death, the release of extracellular traps (ETs), increased endothelial permeability^6^, and aggravated thrombosis^7^. Further, platelets assist leukocytes in adhering to and passing through the endothelium, infiltrating into the affected area^8^. Recently, Paris L. L. *et al*. have reported that porcine liver sinusoidal endothelial cells can phagocytose human platelets in xenotransplantation or xenoperfusion^9–12^. However, little is known about the interplay between endothelial cells (ECs) and platelets in inflammatory circumstances. Since endothelial cells are present in high numbers in micro-circulation and are in intimate contact with platelets, we hypothesized that they might play a role in the clearance of activated platelets in sepsis.

Previous studies have shown that Fc-dependent autoantibodies target the platelet surface complexes GPIIbIIIa and GPIb during platelet clearance^13–15^. Recent studies have also demonstrated that desialylated platelets can be cleared through glycan-lectin binding to hepatic Ashwell Morrell receptors (AMR) or αMβ2 integrins on macrophages^16–19^. Whereas, the mechanism and ligand/receptor by which apoptotic platelets are cleared has not been identified. Phosphatidylserine (PS) is a key ‘eat me’ signal on activated or apoptotic cells for phagocytes^20–22^. However, if elimination of the activated or apoptotic cells is delayed, exposed PS would provide a catalytic surface for the assembly of tenase and prothrombinase complexes^23,24^. In this study, we hypothesized that PS exposure is directly related to procoagulant activity (PCA) on activated platelets and plays a critical role in platelet removal by ECs. The ubiquity and simple structure of PS makes it impractical to synthetize specific antibodies to this phospholipid for identification and/or antagonizing purposes. As an alternative, the milk fat protein lactadherin binds with high affinity and specificity to PS^25^. Lactadherin is a globule membrane glycoprotein secreted by macrophages and ECs, and contains a domain structure of EGF1-EGF2-C1-C2. It binds PS with the C-terminus and phagocyte αVβ3/5 integrins via its RGD motif in the EGF2 domain. Thus, lactadherin acts as a bridge between target cells exposing PS and phagocytes thereby facilitating phagocytosis^26^. Recently, we and others have shown that lactadherin promotes clearance of promyelocytes in acute promyelocytic leukemia and aged erythrocytes and apoptotic cells by angiogenic endothelium^27,28^. Since lactadherin is a readily available specific marker of PS exposure, we examined the role of this molecule in the clearance of activated platelets by ECs in sepsis.

In this study, we hypothesized that platelet disposal by ECs could reduce the formation of platelet heterotypic aggregates and decrease thrombogenicity in sepsis. To gain further insight into the effects of platelet clearance on coagulation, we measured the clotting time, prothrombinase activity, and fibrin formation by platelets before and after exposure to ECs, and examined the benefits of lactadherin-coordinated phagocytosis in ameliorating coagulant abnormality in sepsis.

## Results

### Platelets in sepsis are activated and apoptotic

**Figure 1:**
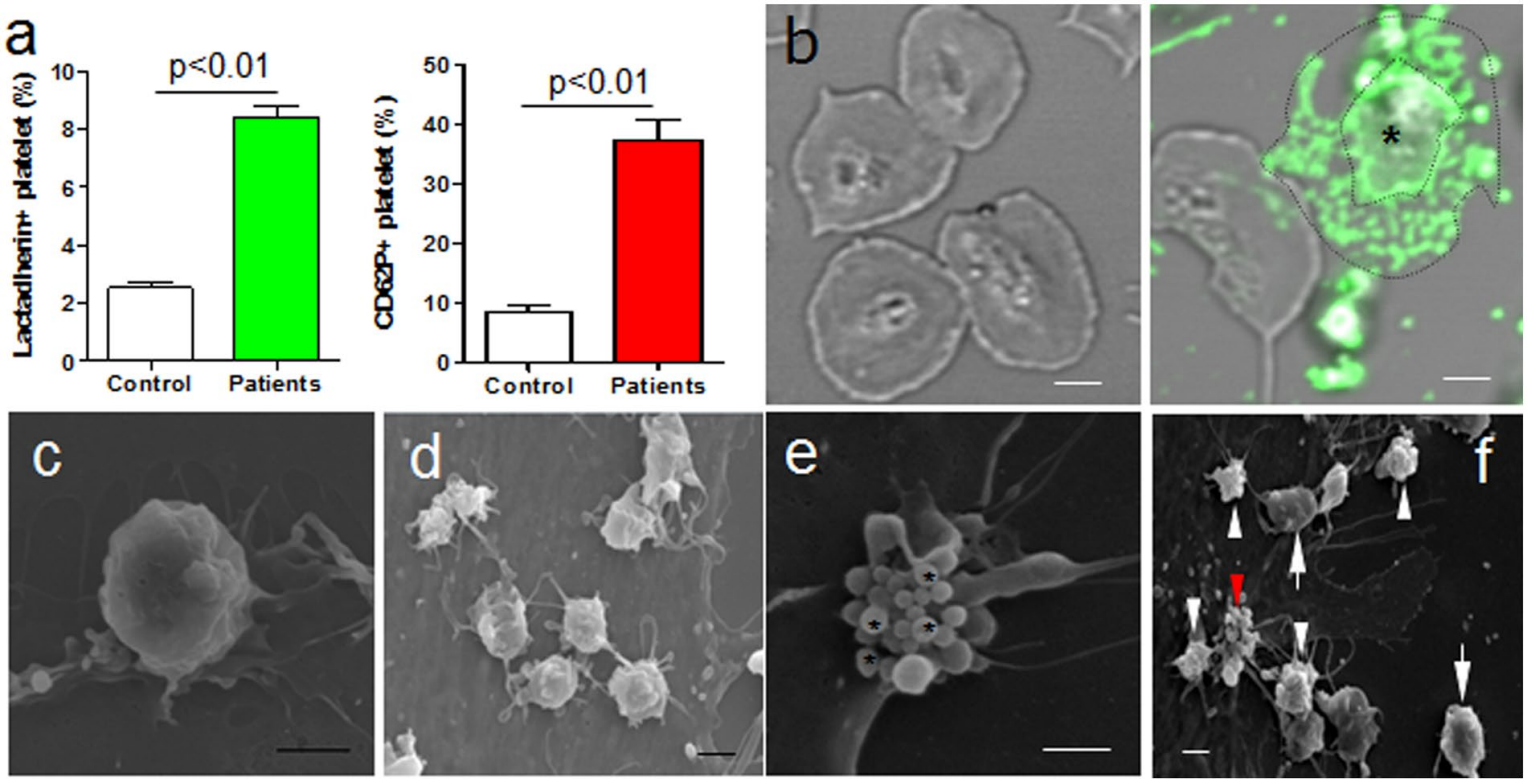
Activation of platelets in sepsis. (**a**) Flow cytometry analysis of PS and CD62P on platelets from healthy controls (n = 27) and patients (n = 27). Data represents mean ± SD. Morphology of platelets from patients (n = 15) were measured by confocal and electron microscopy and some typical forms were presented (**b**–**f**). (**b**) Confocal images showed PS expression on platelets of healthy subjects (left panel) and patients (right panel) stained with Alexa 488 lactadherin. In the patients’ sample, microparticles from one platelet (*) had formed at the margin area located between the distinct outlines. Scanning microscopy showed (**c**) inactivated rounding platelets without pseudopods; (**d**) activated platelets with spreading pseudopods; (**e**) platelet apoptosis with vesicle formation (*); (**f**) quiescent (arrow), activated (white arrowhead) and apoptotic (red arrowhead) platelets in patients. Bars represent 2 μm (**b**–**f**).

Patients'characteristics were present in Tables 1 and 2. Platelets from septic patients showed increased level of surface activation markers identified by lactadherin and CD62P labeling with flow cytometry as compared to control (Fig. 1a). This was further confirmed by confocal imaging of patient platelets showing greater expression of PS and more apoptotic vesicle formation than in healthy controls implying significant activation (Fig. 1b). Additionally, scanning EM (Fig. 1c–f) showed quiescent platelets (Fig. 1c) and platelets were highly activated with extended pseudopodia and ballooning (Fig. 1d,f) that was correlated with microparticle formation (Fig. 1e). The percentage of activated/apoptotic platelets were counted under microscopy: healthy controls (activation 2.5% ± 1.3%; apoptosis 0.6% ± 0.4%) and patients (activation 9.5% ± 3.2%; apoptosis 3.2% ± 1.8%).

### Platelet heterotypic aggregate is a reflection of platelet activation

**Figure 2:**
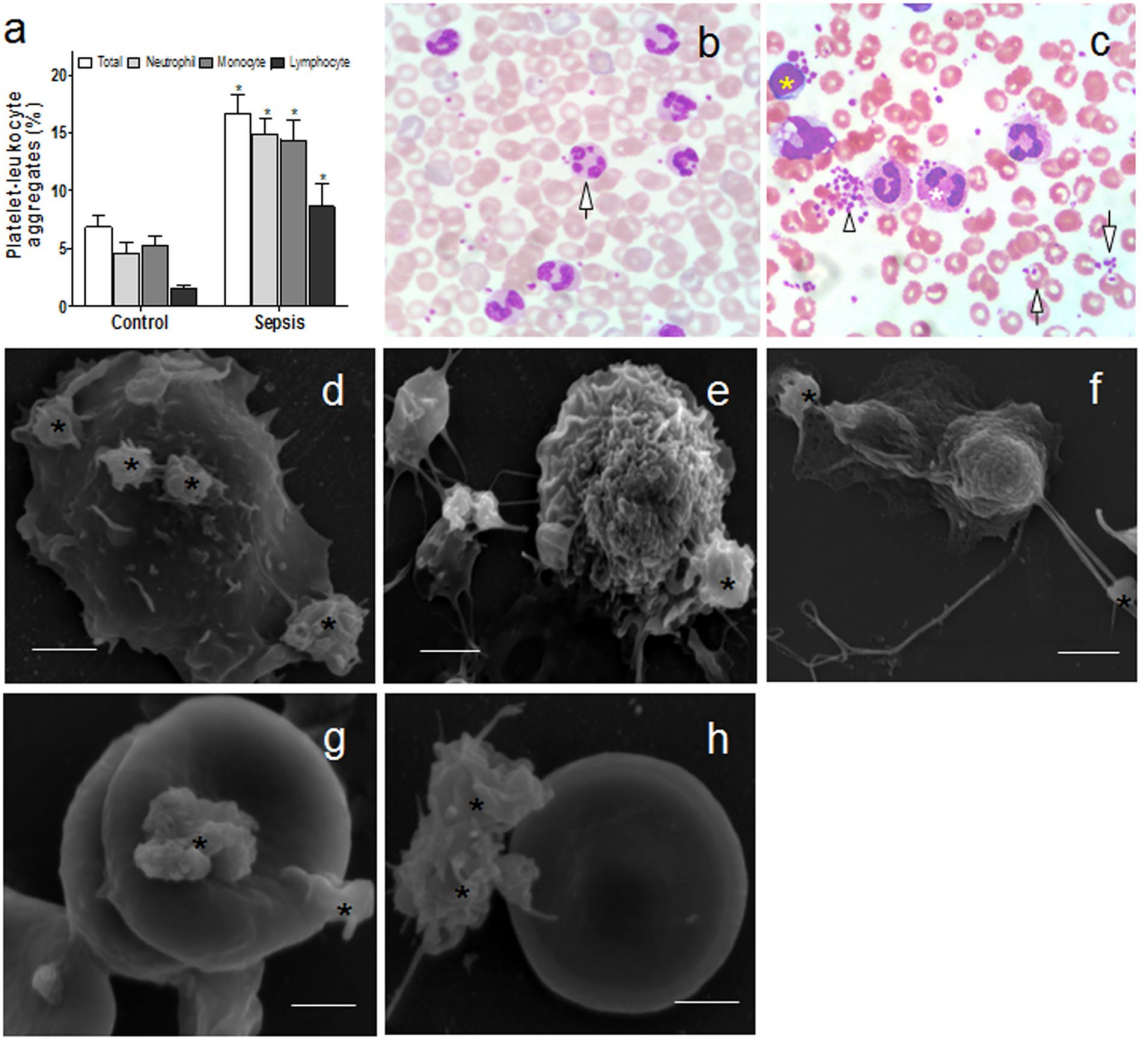
Platelets form heterotypic aggregates with blood cells in circulation. (**a**) The percentage of platelet-leukocyte aggregates (PLA) in healthy controls (n = 27) and patients (n = 27) were measured by flow cytometry. Data represents mean ± SD. *P < 0.05 versus control. (**b**,**c**) Blood smears (n = 15) stained with Hematoxylin-Eosin were observed using a light microscope and typical images of PLA were shown. (**b**) PLA (arrow) in healthy control; and (**c**) several platelets were seen adhered to the neutrophil (* white), lymphocyte (* yellow), and erythrocytes (arrows) in the patient sample. Platelet-platelet aggregation (arrowhead) was also observed. Magnification 40x. Scanning electron microscope images (n = 15): (**d**,**e**) activated platelets (*) anchored on a leukocyte extending pseudopods; (**f**) platelet-leukocyte aggregate releasing extracellular trap-like structures from the nucleus. Single (**g**) and small clusters (**h**) of activated platelets (*) were seen with extended pseudopods or their whole bodies on the membrane of erythrocytes. Bars represent 2 μm.

Formation of various platelet-leukocyte complexes was significantly elevated in septic patients in comparison to healthy controls (Fig. 2a). Based on blood smears, samples from septic patients (Fig. 2c) differed from healthy controls (Fig. 2b) due to the presence of platelet-platelet aggregation as well as primary aggregates of platelets with leukocytes (PLA) and erythrocytes. Electron microscopy further confirmed the presence of compact platelet heterotypic complexes with other blood cells. Activated platelets were observed to adhere to lymphocyte and monocyte-like leukocytes through the spread of their pseudopods (Fig. 2d,e). Interestingly, components similar to extracellular traps appeared to have been released by the PLA (Fig. 2f), suggesting that the PLA may induce inflammatory response through various modalities^29^. Similarly, individual and/or clusters of activated platelets adhered to the surface of the erythrocytes in septic patient blood samples (Fig. 2g,h). Given these results, platelets appear to interact widely with other blood cells, forming both platelet aggregates and platelet-leukocyte/erythrocyte aggregates, indicative of full-blown platelet activation in sepsis.

### Phagocytosis of platelets by ECs

**Figure 3:**
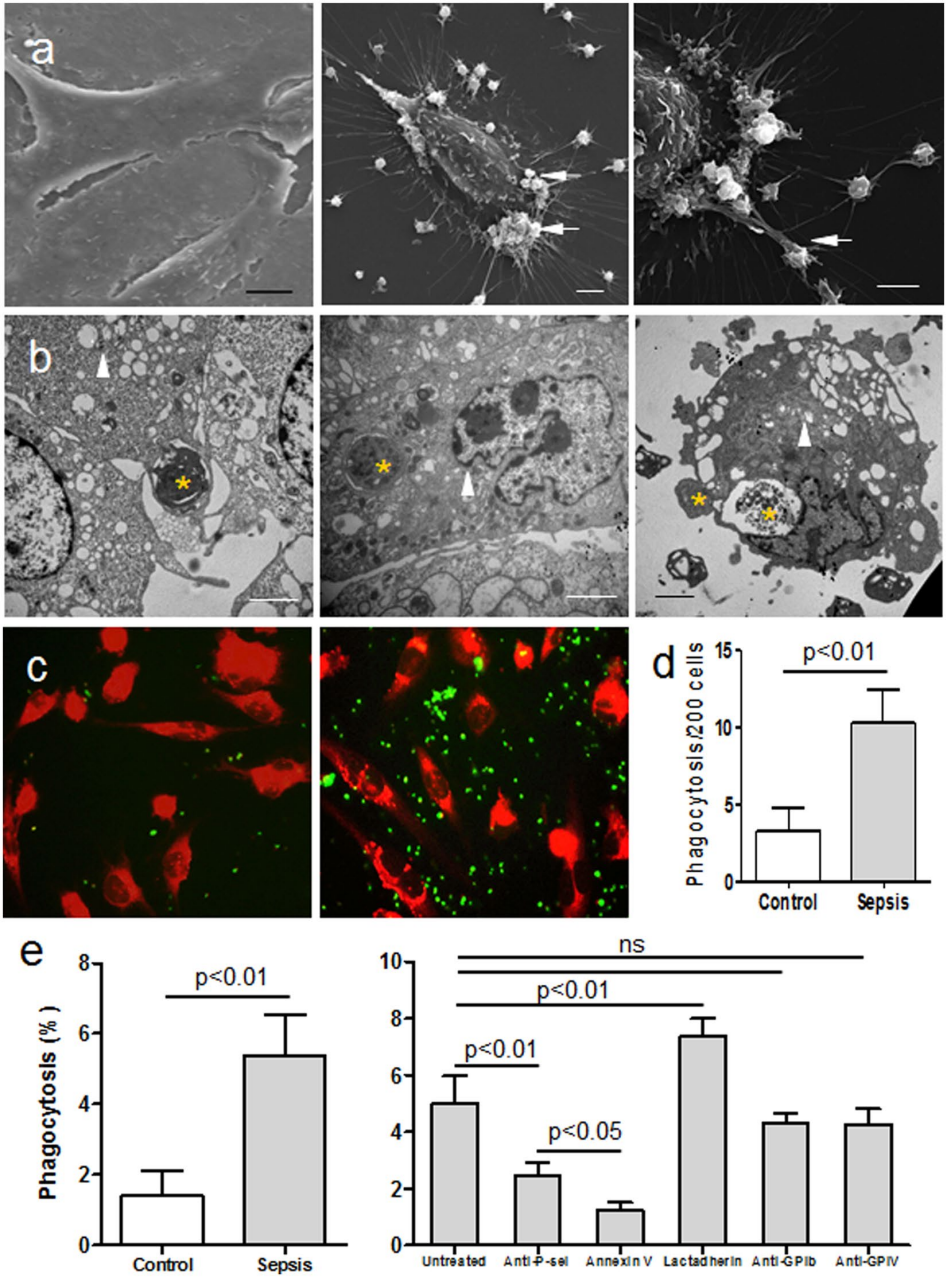
Phagocytosis of platelets by ECs *in vitro*. Platelets of healthy controls (n = 15) and patients (n = 15) were incubated with ECs (triangles) at 37 °C for 30 min and then the mixed cells were fixed for analysis (**a**–**e**). Scanning microscopy images showing that few platelets of healthy subjects adhered to ECs, and ECs did not extend pseudopodia (**a**, left); ECs extended pseudopodia to grasp the stimulated platelets from patients (**a**, middle and right), a proportion of which had anchored on ECs (arrows). (**b**) Transmission microscopy images showing several platelets from patients (*) being trapped, engulfed, and degraded in plasma by ECs. (**c**) CMTPX-labeled ECs (red) and CMFDA-stained platelets (green) from healthy controls (left) and patient samples (right) after co-culture for 30 min were observed using fluorescence microscopy to look for interactions between the two cell types. (**d**) The number of platelets engulfed by ECs (costained yellow) was counted under fluorescence microscopy. Data represents mean ± SD. (**e**) Percentage phagocytosis of platelets from healthy controls and patients were measured by flow cytometry (left). Alternatively, platelets from patients (right) were incubated with ECs in the presence of anti CD62P (20 µg/ml), anti CD42b (20 µg/ml), anti CD36 (20 µg/ml), annexin V (2 nM), or lactadherin (2 nM). Data represents mean ± SD. Bars represent 2 μm (**b**), 5 μm (**a**) and 20 μm (**c**).

*In vitro* experiments and scanning EM of healthy platelets co-cultured with ECs, showed few platelets adhering to ECs and that the ECs did not extend pseudopods (Fig. 3a, left). However, platelets of septic patients actively attached to the surface of cultured ECs either individually or in clusters and were partially engulfed by endothelial pseudopods (Fig. 3a **middle and right**). Transmission EM also revealed that the platelets were encircled, trapped, and ultimately endocytosed by the ECs. Platelets could then be seen as fragments present in large vacuoles in the ECs (Fig. 3b). These phagocytic vacuoles did not appear in ECs cultured in the absence of platelets or in those that were incubated with control platelets derived from healthy donors (data not shown). These findings revealed that ECs can bind and endocytose activated platelets obtained from septic patients, but not quiescent platelets of healthy individuals (Fig. 3c,d).

**Figure 4:**
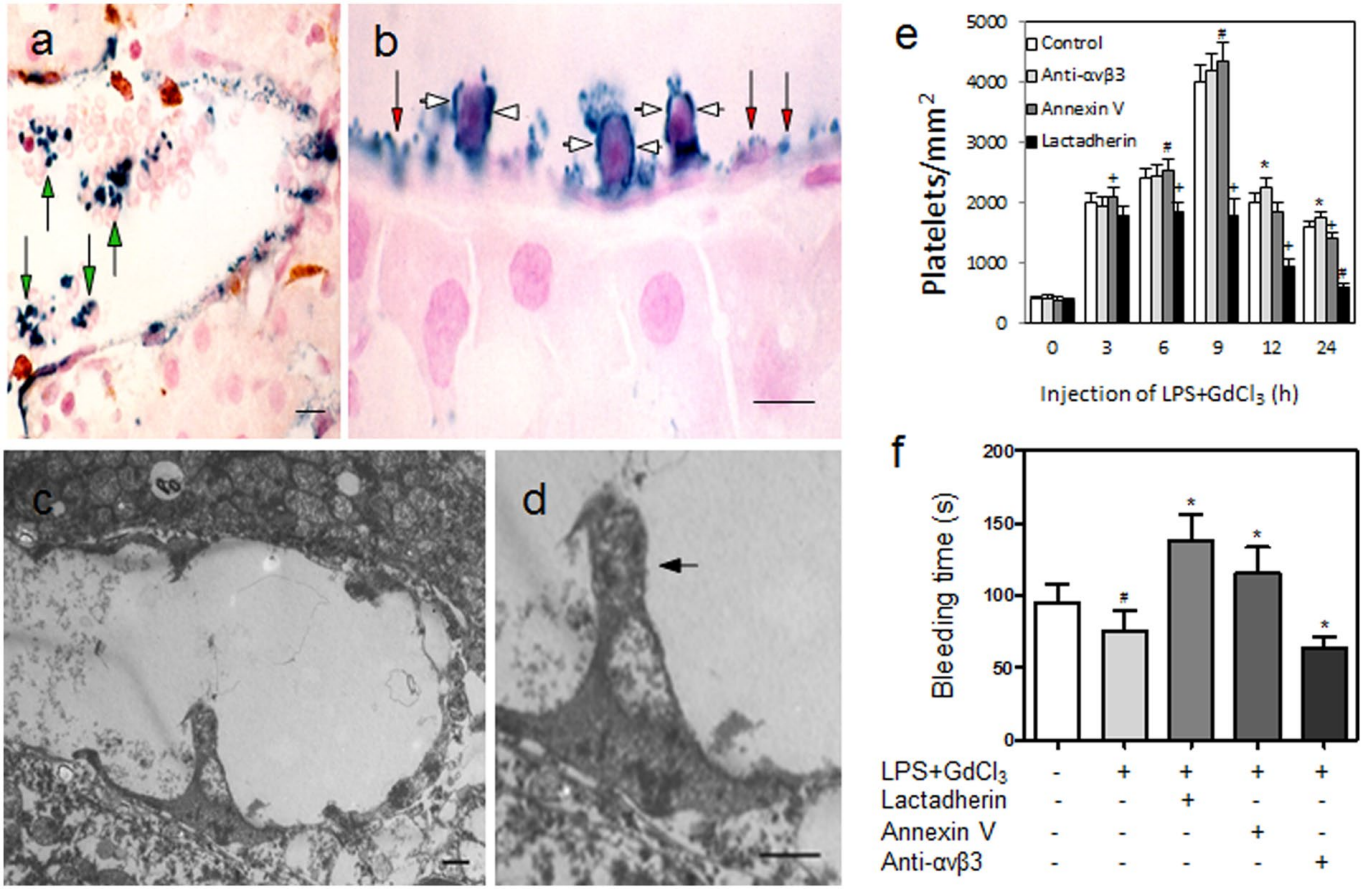
Phagocytosis of platelets by ECs *in vivo*. Rats were injected with LPS and the livers were taken at 6 h after perfusion fixation (**a**–**d**). (**a**,**b**) Platelets were immunolabeled with anti-P-selectin antibody (blue) and counterstained with nuclear fast red (red). Many platelets expressing P-selectin were found adhering on ECs in the portal vein (red arrows) and also formed aggregates with leukocytes (white arrows) and erythrocytes (green arrows) in the liver. (**c**,**d**) Transmission electron microscopy images further confirmed engulfment and partial digestion (arrow) of the platelets by the ECs *in vivo*. Bars represent 2 μm (**c**,**d**) and 10 μm (**a**,**b**). Mice were also injected with LPS to induce septic conditions and GdCl_3_ to block the function of Kupffer cells in the liver (**e**,**f**). (**e**) Platelet number in the liver was counted and present as number/mm^2^ with various treatments in different time points. Each value is expressed as mean ± SD of 10 mice. *# + P < 0.05 versus control. (**f**) Septic mice were pretreated with lactadherin, annexin V, or anti-αvβ3 antibody, and tail-bleeding time was evaluated. Each value was expressed as mean ± SD of 10 mice. *P < 0.05 versus mice only treated with LPS and GdCl_3_, ^#^P < 0.05 versus untreated mice.

The elevated platelet PS exposure was paralleled by an increased percentage of patient platelets that were phagocytosed compared with healthy controls (Fig. 3e, **left**). Blocking PS with annexin V or P-Selectin with anti CD62P antibody attenuated phagocytosis significantly (Fig. 3e, right and the supplementary information). In contrast, treatment with anti GPIb or anti GPIV antibodies had no effect. Addition of lactadherin, which acts as a bridge between exposed PS on apoptotic cells and integrins on phagocytes, increased EC phagocytosis by approximately 2-fold. The amount of phagocytosis was negatively correlated with the number of platelets observed in the liver. Injection of annexin V led to increased platelets counts in the liver while promoting PS-mediated engulfment with lactadherin led to a significant decrease in these platelet counts (Fig. 4e). However, while promoted endocytosis induced an efficient clearance of activated platelets, there was no significant decrease in overall platelet count. Thus the main action of endocytosis was to avoid further aggregation, and thereby reduce the risk of thrombocytopenia.

To investigate the elimination of platelets by ECs *in vivo*, we used our previously developed animal models^30,31^ that show the interactions between platelets, leukocytes, and EC. Both rats and mice were injected with LPS to mimic septic conditions. Immunohistochemistry of livers revealed that platelets adhered to EC and formed aggregates with leukocytes on the surface of EC and in the lumen (Fig. 4a,b). Transmission EM further confirmed that ECs had engulfed and partially digested the platelets (distinguished by size and granules) (Fig. 4c,d). Pretreatment with GdCl_3_ depleted the Kupffer cells as our previous study described^31^. Injection of LPS induced platelet accumulation in the liver in a time-dependent manner (Fig. 4e). Pretreatment with annexin V, which binds PS on activated platelets, blocked platelet clearance and led to increased platelet count in the liver. However, blocking PS with annexin V also prolonged bleeding time (BT) (Fig. 4f). Additionally, inhibition of αvβ3 integrin on ECs *in vivo* decreased the clearance of platelets and resulted in a rise in platelet count in the liver 6-hour post LPS injection. This delayed clearance of activated platelets and shortened BT (Fig. 4f).

### Platelet clearance by ECs results in fewer PLA formation

**Figure 5:**
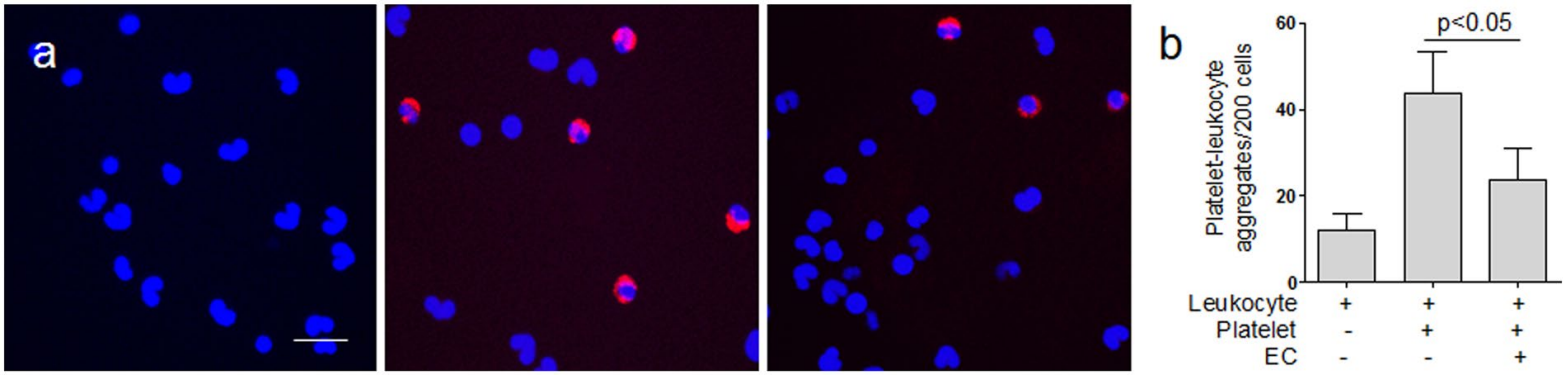
Platelet phagocytosis by ECs reduces PLA formation. (**a**) Platelets of patients (n = 15) were collected and labeled with Alexa Fluro 647-conjugated CD41a (red). Platelets before (middle) or after (right) engulfment by ECs were co-cultured with leukocytes (stained with DAPI, blue) from healthy subjects. Immunofluorescence showed that PLA formation was decreased after platelet phagocytosis by ECs. Untreated leukocytes from healthy individuals were used as control (left). Bars represent 20 μm. (**b**) The number of PLA was counted under immunofluorescence before and after platelets were incubated with ECs. The number of PLA in untreated leukocytes from healthy individuals was used as control. Data represents mean ± SD.

Furthermore, platelets were pre-incubated with ECs *in vitro*, and then the platelets that had not been endocytosed were detached and harvested after treatment with EDTA. These platelets were then incubated with leukocytes from healthy individuals. Platelets and leukocytes from healthy individuals were also incubated as control. We found that platelets from patients pre-incubated with ECs formed markedly fewer PLA (Fig. 5), suggesting that activated platelets were cleared and formed fewer PLA with leukocytes. It is important to remove activated platelets for diminishing aggregate formation between surviving platelets and leukocytes that could impede circulation and cause ischemia in septic patients. Tables 1 and 3.

**Table 1:** Septic patients’ characteristics.

Variables	Control (n = 60)	Sepsis (n = 60)
Age (years)	52 ± 6	53 ± 11
Sex (Male/Female)	35/25	35/25
Vital signs		
Temperature (ÿC)	36 ± 0.5	38.5 ± 1.1
Respiratory Rate (bpm)	16 ± 3	22 ± 5
Heart Rate (bpm)	74 ± 7	82 ± 14
SBP (mm Hg)	96 ± 7	115 ± 12
DBP (mm Hg)	75 ± 13	73 ± 18
MAP (mm Hg)	85 ± 13	90 ± 21
Glasgow Coma Scale score	15	14 ± 1.2
Laboratory results		
WBC (109/L)	6.5 ± 3.7	13.2 ± 6.3*
Platelet (109/L)	225 ± 34	196 ± 85
PO_2_	85 ± 11	75 ± 12
PO_2_/FiO_2_ (mm Hg)	413 ± 22	325 ± 54
Bilirubin (mg/dL)	0.4 ± 0.1	0.9 ± 0.3*
Creatinine (mg/dL)	0.5 ± 0.2	1.4 ± 0.6*
Bacteria Culture +	—	39
Gram+/−	—	27/12
Coagulation parameter		
PT (s)	13 ± 4.3	16.5 ± 11.4
APTT (s)	26.8 ± 5.2	33.5 ± 14
Fibrinogen (g/L)	3.2 ± 0.7	2.8 ± 1.7
D-dimer (g/L)	0.9 ± 0.5	8.1 ± 5.6*
DIC	—	2

Values given in mean and SD. ^*^P < 0.05 vs. control. Abbreviations: SBP, systolic blood pressure; DBP, diastolic blood pressure; MAP, mean arterial pressure; WBC, white blood cell; PT, prothrombin time; APTT, activated partial thromboplastin time; DIC, disseminated intravascular coagulation.

**Table 2:** Characteristics of Septic patients developing DIC.

	Patient 1	Patient 2
Admission		
Platelet (10^9^/L)	167	203
PT (s)	16.3	14
APTT (s)	33	29
Fibrinogen (g/L)	2.8	3.6
D-dimer (g/L)	5.5	3.8
DIC occurance		
Day (after admission)	3	5
Platelet (10^9^/L)	9.5	12.3
PT (s)	24.3	20.8
APTT (s)	42	37
Fibrinogen (g/L)	0.4	0.7
D-dimer (g/L)	12.6	11.3

Abbreviations: PT, prothrombin time; APTT, activated partial thromboplastin time; DIC, disseminated intravascular coagulation.

### Coagulation disorder in septic patients

**Figure 6:**
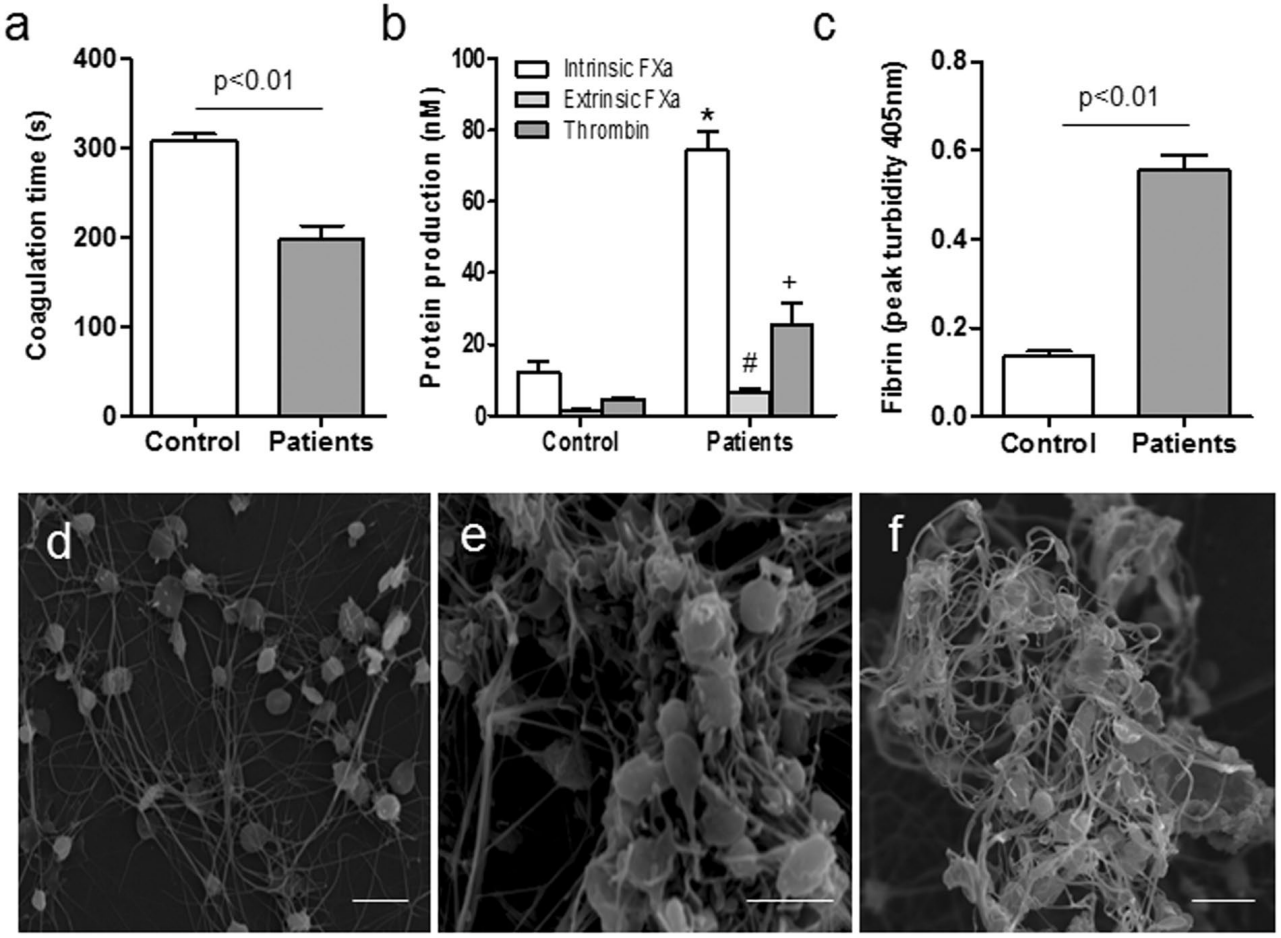
Coagulation dysfunction in septic patients. Platelets from patients (n = 15) were tested for (**a**) coagulation time, (**b**) intrinsic FXa, extrinsic FXa, thrombin formation, and (**c**) fibrin clot turbidity. Platelets from healthy individuals (n = 15) were used as controls. Scanning microscopy images of hypercoagulant platelets from patients showed (**d**,**e**) fibrin net and (**f**) microthrombus formation. Data represents mean ± SD; *^#^ + P < 0.05 versus healthy controls. Bars represent 5 μm.

Platelets derived from patients supported reduced coagulation times in comparison to those from healthy controls (Fig. 6a). Notably, elevated FXa generation correlated with a 4-fold increase in thrombin formation in patients compared to controls (Fig. 6b). Furthermore, this increase in thrombin resulted in significantly increased fibrin production in patients (Fig. 6c). Large amounts of microthrombi filled with platelets were also observed in septic samples (Fig. 6d–f), indicating that coagulation dysfunction occurs in septic patients in addition to hypercoagulability and greater fibrin formation.

### Lactadherin-enhanced phagocytosis reduces PCA and fibrin formation in septic patients

**Figure 7:**
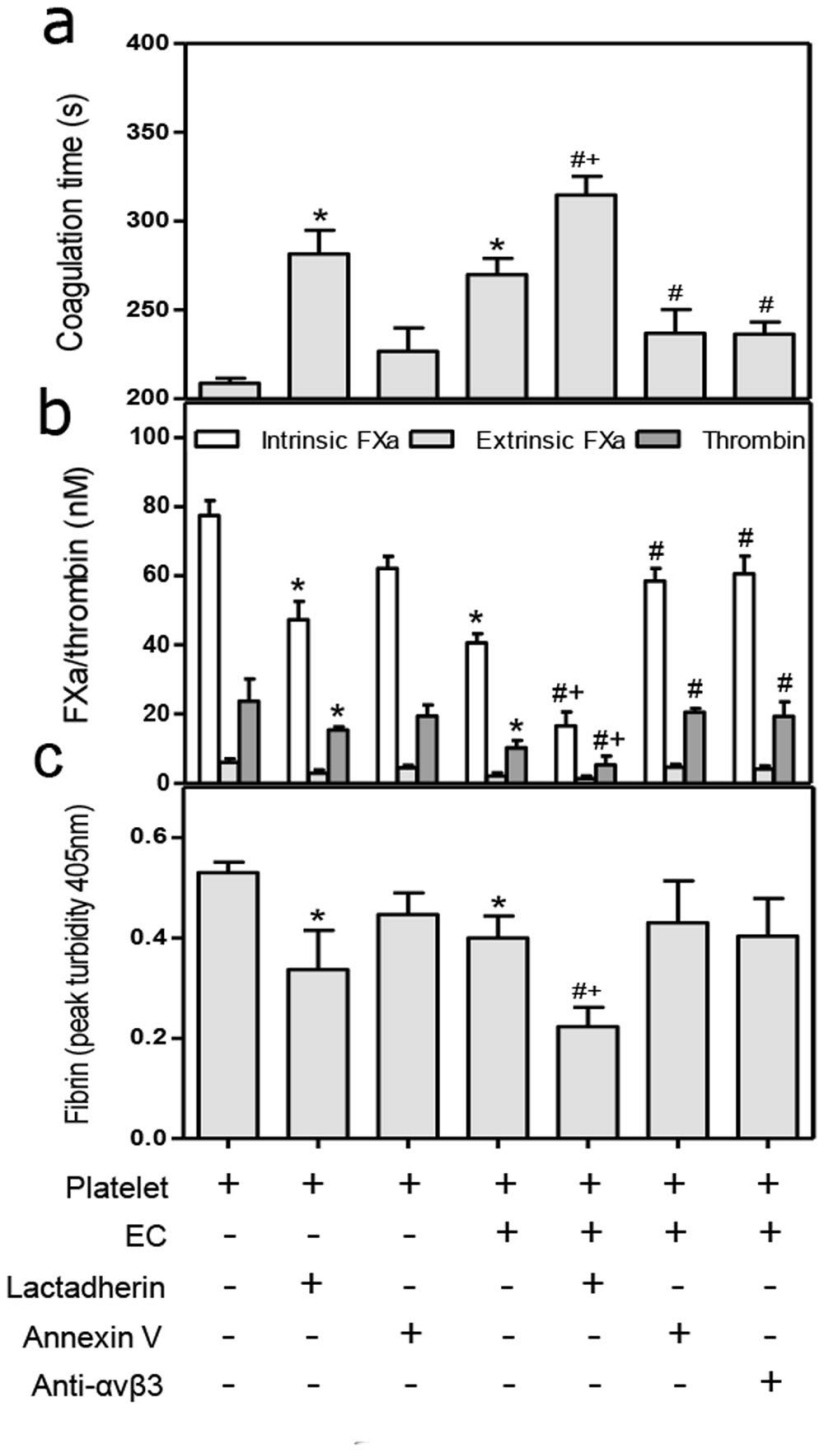
Effect of lactadherin-mediated phagocytosis on procoagulant activity and fibrin formation. Platelets from patients (n = 10) were collected. They were untreated, treated with lactadherin or annexin V, or incubated with ECs for 1 h in the presence or absence of lactadherin, annexin V, or anti-αvβ3 antibody. The untreated or treated platelets were for further evaluations of (**a**) coagulation time; (**b**) intrinsic and extrinsic FXa and thrombin generation; (**c**) and fibrin turbidity which was measured as terminal A_405_ at half an hour. Platelets without any treatment were used as control. *P < 0.05 versus control; ^#^P < 0.05 versus Platelet ( + ) ECs ( + ); + P < 0.05 versus Platelet ( + ) lactadherin ( + ). Each value represents mean ± SD.

The implication of platelet clearance by phagocytosis motivated us to explore how this effect influenced PCA and fibrin formation. Elimination of abnormal platelets from patients by ECs *in vitro* increased coagulation time and decreased intrinsic FXa and thrombin production by the remaining platelets (Fig. 7). Inhibiting αvβ3 integrin on ECs led to a marked rise in coagulation complexes compared with ECs alone. Blocking PS with annexin V diminished the platelet engulfment, increasing FXa and thrombin generation and decreasing clotting time (Fig. 7b). These results were in accordance with the reduced platelet PCA seen in patients in remission (Table 3). Compared with use of HUVECs alone, a combination of lactadherin and ECs resulted in up to 50% inhibition in fibrin generation (Fig. 7c), the largest decrease in coagulation complex formation, and an increase in clotting time to near normal (Figs 6a and 7a). These findings indicate that administration of lactadherin may work in a cooperative manner with patient ECs to significantly decrease activated platelet-induced formation of PCA and fibrin.

**Table 3:** Platelet activation in patients during and after sepsis.

Platelet activation	Onset	Remission
PS (%)	8.2 ± 2.6	5.4 ± 2.3^*^
P-selectin (%)	49.7 ± 9.3	24.6 ± 2.7^*^
PLA (%)	16.8 ± 2.3	8.2 ± 1.2^*^
Coagulation time (s)	208.8 ± 4.7	291 ± 16^*^
Thrombin (nM)	23.1 ± 5.4	10.9 ± 2.1^*^

Values given in mean and SD. ^*^P < 0.05 vs. patients onset. PS, phosphatidylserine; PLA, platelet-leukocyte aggregates.

## Discussion

Phagocytosis of activated platelets by dedicated phagocytes has been well documented. However, leukocytes also undergo apoptosis and may be overwhelmed and unable to clear all of the activated and apoptotic platelets in sepsis. In our study, we show that activated platelets are tethered, endocytosed, and degraded by ECs in sepsis. Additionally, ECs treated with septic serum *in vitro* trapped more activated platelets than those treated with healthy serum. While phagocytosis of platelets promoted EC activation, it did not impact EC survival^32^. Phagocytosis of platelets was decreased by either blocking PS on platelets with annexin V or by blocking the αvβ3 integrin on ECs. In contrast, lactadherin can simultaneously bind to platelet PS via its C2-terminus and to αvβ3 integrins on ECs via its RGD motif in the EGF2 domain^33^ and may therefore facilitate engulfment of platelets. Previous studies have shown that lactadherin similarly promotes removal of apoptotic erythrocytes, lymphocytes, and microparticles by macrophages^34–36^ consistent with our hypothesis. In this study, we have shown that lactadherin binding to PS significantly enhances phagocytosis of platelets by the ECs. These results suggest that PS plays a critical role in platelet engulfment and removal. Of note, enhanced endocytosis of platelets with addition of lactadherin doesn’t cause thrombocytopenia, suggesting that lactadherin is selective for activated platelets rather than quiescent ones and that activated platelets are a small subset of total platelets. Our demonstration of phagocytosis of activated platelets not only provides additional insight into platelet-EC interaction but also reveals a novel mechanism for platelet clearance in sepsis.

We also evaluated the roles of other receptors in platelet removal. Our results showed that inhibition of P-selectin on platelets leads to a marked reduction in platelet clearance while blockade of GPIV (CD36) has only a slight effect on phagocytosis of platelets by ECs. GPIb plays an important role in tethering and rolling of platelets on the EC^37^, and recent studies show that GPIb sialyation is involved in platelet clearance mediated by the Ashwell Morrell receptor (AMR) or Asialoglycoprotein receptor-1 (ASGR1)^6,7^. In this study, inhibition of GPIb on platelets in septic patients did not significantly affect platelet clearance, which is consistent with the study by Norma Maugeri *et al*.^38^. However, the alterations of GPIb structure on human platelets after desialylation in sepsis and the interactions between apoptotic and glycan-lectin mediated platelet clearance need to be further studied. Furthermore, thrombospondins (TSP) associated with CD36 antigens promote platelet adherence to phagocytes^37^. Thus the role that TSP plays in platelet clearance by ECs will be evaluated in further studies.

Platelet-leukocyte interactions play an important role in thrombo-inflammatory injury by actively contributing to intravascular inflammation, leukocyte recruitment to inflamed sites, and the amplification of the procoagulant response^39^. In our study, we showed that septic platelets associated with activated leukocytes, forming platelet-leukocyte complexes, implying intense platelet activation and severe inflammatory response^40,41^. Furthermore, leukocytes that were bound to activated platelets release an extracellular trap-like substance that may have procoagulant and prothrombotic effects. Interestingly, we also found many instances of platelets adhering to erythrocytes, indicating activation of all blood cell types in circulation. Given the aggregates of platelets and other blood cells in circulation designated as platelet-hemocyte complexes, we propose that such complexes may play an important role in the induction of inflammation, coagulation, and thrombosis in sepsis and in other conditions where such complexes would be commonly observed. Furthermore, we also demonstrate that clearance of activated platelets by ECs also contributes to inhibition of PLA formation, consistent with the decline of PLA generation when patients go into remission. Reduction of PLA may thus prevent excessive inflammatory response and alleviate the burden of coagulation, analogous to antiplatelet treatment mediating suppression of platelet-neutrophil aggregate formation and neutrophil activation^42,43^.

In our study, platelet counts are in the normal range in septic patients and we suggest that in the early phase of sepsis, the platelet counts may not change significantly and are in the normal range until the stage of platelet consumption in DIC^44–46^. However, the activation of platelets in the early phase of sepsis indeed exists which contributes to the altered procoagulant activity of platelets. We observed significantly increased PS exposure on platelets and higher platelet-derived microparticle count in patient blood compared to samples from healthy volunteers. Microscopy analysis of blood samples further showed the presence of apoptotic platelets with microparticles, indicating that platelets are damaged during infection. As a consequence, patients showed a propensity for thrombosis with fibrin and microthrombus formation. The PCA of activated platelets significantly decreased when PS was specifically blocked with lactadherin. Moreover, we found that phagocytosis of PS-exposed platelets by ECs prolongs coagulation time, and diminishes the formation of procoagulant enzyme complexes by approximately 50% (Fig. 7b), which inhibits fibrin production by one-third in comparison to values prior to phagocytosis. Lactadherin enhances EC phagocytosis and further reduces PCA and fibrin formation of activated platelets, contributing to better amelioration of coagulation abnormality than either treatment alone. Thus, we indicate that in case of early and prompt clearance of activated platelets in sepsis, the normal platelet counts will not have significant change and these findings have implications for treatment and prevention of DIC in sepsis.

Antiplatelet treatment has been suggested as a novel strategy in sepsis^47^, and we speculate that promoting efficient removal of activated and apoptotic platelets could further improve patient outcomes. A large decline in PS exposure on platelets, associated platelet PCA, and PLA formation is seen in patients in remission, which could be attributed to the elimination of abnormal platelets. Therefore, clearance of activated platelets earlier in the disease process could hasten recovery of homeostasis in circulation by eliminating catalytic platforms for the coagulation pathway, protecting blood cells from excessive activation, and restoring their normal function. Endothelium, at least in part, contributes to platelet disposal and may further improve the hypercoagulable status in inflammation. It is noteworthy that PS-mediated and lactadherin-strengthened platelet engulfment may modify coagulopathy, and thus provide a new modality for treatment of septic clotting disorders.

## Materials and Methods

### Patients

Peripheral venous blood was obtained upon informed consent from 60 healthy subjects who had not received any medication in the past 2 weeks, and from 60 patients diagnosed with sepsis (not including severe sepsis and sepsis shock) between Dec. 2012 and Apr. 2017. The diagnosis of sepsis was on the basis of documented or suspected infection, presence of systemic signs and symptoms of inflammation and potential organ dysfunction (Sequential [Sepsis-related] Organ Failure Assessment (SOFA))^48,49^. Exclusion criteria were age <18 years, cardiovascular disease, diabetes, liver or renal dysfunction, cancer, platelets and/or blood coagulation disorders and prior treatment with anticoagulant and/or antiplatelet therapy. DIC was diagnosed as the diagnostic criteria^50,51^. Study was performed with the approval of the Ethics Committee of Harbin Medical University in accordance with the Declaration of Helsinki.

### Reagents

Endothelial cell medium was purchased from AllCells (Emeryville, CA, USA). Bovine serum albumin (BSA), poly-d-lysine, ethylenediaminetetraacetic acid (EDTA), lipopolysaccharide (LPS), Triton X-100, 4′,6-diamidino-2-phenylindole (DAPI), 3,3-diamino-benzidine tetrahydrochloride (DAB), gadolinium chloride (GdCl_3_) and annexin V were obtained from Sigma-Aldrich (St Louis, MO, USA). 0.25% Trypsin-EDTA was from Gibco (Grand Island, NY, USA). CellTracker Green CMFDA and CellTracker Red CMTPX were from Invitrogen (Carlsbad, CA, USA). Lactadherin was prepared in our laboratory. Human factors Va, VIIa, VIII, IXa, X, Xa, prothrombin and thrombin were all from Haematologic Technologies (Burlington, VT, USA). The Chromogenix substrates S-2238 and S-2765 were from DiaPharma Group (West Chester, OH, USA). Monoclonal antibodies against CD42b (glycoprotein Ib, clone HIP1), CD62P (P-selectin, clone AK-4), CD41a (clone HIP8), CD45 (clone H130), CD36 (glycoprotein IV, clone CB38) were from Becton Dickinson Biosciences (San Jose, CA, USA). Monoclonal antibody against αvβ3 integrin was from Abcam (ab7166), Labome (clone LM609), and polyclonal antibody from Bioss (bs1310R). Alexa Fluro 488 or 647-conjugated CD41a and Alexa Fluro 647-labeled CD45 were prepared in our laboratory. FITC-labeled monoclonal antibody against CD62P was from Abcam (ab6632, Cambridge, MA, USA).

### Cell preparation

Peripheral venous blood was drawn with a 21-gauge needle and collected into a 5-ml tube containing 3.8% sodium citrate. Specimens were centrifuged for 15 minutes (min) at 200 g at room temperature (RT); platelet-rich plasma (PRP) was isolated and diluted with modified Tyrode’s buffer (137 mM NaCl, 2.7 mM KCl, 11.9 mM NaHCO_3_, 0.42 mM NaH_2_PO_4_, 1 mM MgCl_2_, 5.5 mM glucose, 5 mM HEPES and 0.35% BSA, pH 7.4) supplemented with 1 mM EDTA. Platelets were pelleted by centrifugation at 1000 g for 5 min and resuspended in Tyrode’s buffer. Platelet count was calculated with a blood cell counter (Cellometer, Nexcelom, America). Leukocyte-rich layer was collected and after hypotonic lysis of erythrocytes; the suspension was centrifuged at 300 g at RT for 5 min. Leukocyte pellet was washed and then resuspended in 1 ml Tyrode’s buffer for use.

Human umbilical vein ECs (HUVECs) from AllCells (Emeryville, CA, USA) were cultured in endothelial cell medium containing 10% FBS, 1% endothelial cell growth supplement and antibiotics at 37 °C in a 5% CO_2_ humidified atmosphere. Cells from 2–6 passages were used for experiments.

### Co-incubation assay

Platelets (4 × 10^7^) were incubated with ECs (2 × 10^5^) in 12-well culture plates for 30 min at 37 °C in serum-free medium containing CaCl_2_ (1 mM). In some cases, platelets were pretreated with lactadherin (2 nM) or annexin V (2 nM) and ECs were pretreated with anti-αvβ3 (5 µg/ml) at RT for 15 min. After incubation, the mixed cells were washed and treated with 0.25% trypsin-EDTA for 2 min at 37 °C to promote detachment and remove extracellular adherent platelets^52^. Alternatively, platelets (2 × 10^6^) with or without prior engulfment by ECs were added to leukocyte suspension (1 × 10^5^) and incubated for 30 min.

### Flow cytometry

To quantify PS and P-selectin expression, platelets or HUVECs were adjusted to 1 × 10^6^ cells/ml and incubated with FITC-anti-P-selection (5 nM) and Alexa 647-labeled lactadherin (2 nM) for 15 min in the dark at RT. Cells were analyzed on a flow cytometer (FACSA Canto II, Becton Dickinson, USA).

For *in vitro* EC-platelet phagocytosis and inhibition assays, platelets stained with 3 µM CMTPX (excitation 586/emission 613, red) were incubated with ECs labeled with 1 µM CMFDA (excitation 492/emission 516, green) as described in “Coincubation assay”. The mixed cells were collected, washed and incubated with 0.25% trypsin-EDTA for 2 min at 37 °C to promote detachment and remove any surface bound platelets^52^. For inhibition assays, platelets were preincubated with anti CD62P, anti CD42b, anti CD36, annexin V, or lactadherin followed by co-cultured with ECs at 37 °C for 30 min. Phagocytosis was determined by the percentage of red fluorescence (CMTPX)-positive CMFDA (green) ECs by FACS Canto II flow cytometer^27^.

Analysis of PLA has been described previously with some modifications^53^. Percentages of platelet-conjugated leukocytes in the total leukocyte, monocyte, lymphocyte, and neutrophil populations were obtained. Briefly, 50 µl of leukocyte suspension were added to Alexa Fluro 488-conjugated CD41a (2 µg/ml) and Alexa Fluro 647-conjugated CD45 (2 µg/ml) for 15 min at RT in the dark. The cells were then washed, resuspended in Tyrode’s buffer, and analyzed immediately by flow cytometry.

### Confocal microscopy

Platelets from healthy subjects and patients were incubated with Alexa Fluro 488-labeled lactadherin (4 nM) to detect PS exposure. Cells were washed to remove unbound protein and evaluated immediately. The samples were excited with the 488 nm emission line of a krypton-argon laser, and narrow bandpass filters were utilized to restrict emission wavelength overlap. Images were captured with Zeiss LSM 510 Meta confocal microscope (Carl Zeiss Jena GmbH, Jena, Germany).

### Electron microscopy

For scanning electron microscopy (EM), whole blood smear or ECs incubated with platelets were prepared on glass coverslips. Samples were treated with 2.5% glutaraldehyde fixative and stored at 4 °C until processed. Following several rinses in 0.1 M Na-cacodylate HCl buffer, samples were then fixed with 1% OsO_4_ before being dehydrated in various concentrations of ethanol. A layer of platinum, approximately 10 nm thick, was sprayed on the samples. Images were examined with a S-3400N Scanning Electron Microscope (Hitachi Ltd., Tokyo, Japan) using an ultra-high-resolution mode. The qualification of activated/apoptotic platelets was measured as previously with some modifications^54^. Briefly, 200 cells were randomly counted and the percentage of activated/apoptotic platelets was analyzed.

For transmission microscopy, patient whole blood, livers of septic rats, or ECs that had been previously incubated with platelets were double fixed in 2.5% glutaraldehyde and 1% OsO_4_ before washing, dehydration and embedding. Conventional thin sections were prepared with Reichert-Jung Ultracut Ultramicrotome (Leica, Vienna, Austria). Images were observed under a H7650 transmission electron microscope (Hitachi Ltd., Tokyo, Japan).

### Fluorescence microscopy

Platelet-leukocyte complexes was detected by modified method as previously described^38^. Briefly, the mixture of platelets and leukocytes was seeded in 12-well plates coated with poly-d-lysine for 1 h at 37 °C in a 5% CO_2_ atmosphere. They were subsequently fixed in 3.7% formaldehyde for 1 h at 4 °C and blocked with 10% BSA. For fluorescence staining, platelets were labeled with Alexa Fluro 647-conjugated CD41a (2 µg/ml), followed incubation with DAPI (500 ng/ml) to detect leukocytes. Specimens were washed and identified with fluorescence microscope (Leica, DM400B, Germany). The qualification of PLA was measured as previously with some modifications^54^. Briefly, 200 cells were randomly counted and the percentage of PLA (costained with red and blue) was analyzed.

Engulfment was analyzed using a previous method with some modifications^27^. Briefly, ECs were seeded on glass coverslips coated poly-d-lysine in 12-well culture plates. In some cases, ECs were pretreated with 10% septic serum for 6 h and then were washed three times with Tyrode’s buffer to remove the residual serum. ECs were labeled with 1 µM CMTPX, and then they were co-incubated with 3 µM CMFDA-stained platelets as described in “Coincubation assay”. The mixed cells were fixed in 3.7% formaldehyde and analyzed immediately. Platelets phagocytosed by ECs were co-stained with both CMFDA (green) and CMTPX (red). The qualification of englufed platelet was measured as previously with some modifications^54^. Briefly, 200 cells were randomly counted and the percentage of engulfed platelets (costained yellow) was analyzed.

### Immunohistochemistry

To observe the distribution and phagocytosis of platelets *in vivo*, the following experiments were performed as described previously with some modifications^31^. Briefly, 7- to 9-week-old male Wistar rats were injected with LPS (0.1 mg/kg intravenously [IV]) or sterile saline as control, and perfusion fixed at 6 h after injection. Samples of the liver were taken for microscopic examinations. The livers of the control and treated animals were perfused through the portal vein with a peristaltic pump at a rate of 10 ml/min. Perfusion consisted of saline for 20 sec followed by phosphate-buffered 4% paraformaldehyde for 2 min. After perfusion, slices of liver 3-mm thick were immersed in fixative for 6 h at 4 °C, then embedded in paraffin. Selected sections (4 µm) of the control and treated liver were immunostained by streptavidin- biotinperoxidase complex method. These sections were pretreated with 0.3% H_2_O_2_ in methanol to block endogenous peroxidase activity and were then incubated with normal goat serum (1:5 dilution). The sections were then incubated overnight with primary Abs (diluted 1:1000), rinsed in phosphate-buffered saline (PBS) containing 0.03% Triton X-100 and then incubated for 30 min with either biotinylated goat anti-mouse Ig (1:600 dilution) or biotinylated goat anti-rabbit Ig (1:800 dilution, for anti–P-selectin). Sections were then rinsed again in PBS, and finally, incubated for 30 min with peroxidase-conjugated streptavidin (1:300 dilution). All Abs were diluted in 0.1 mol/L PBS containing 0.03% Triton X-100 and 1% bovine serum albumin (BSA), and all steps were performed at room temperature. Tissue peroxidase activity was visualized after a 5-minute exposure to 0.025% 3,3-diamino-benzidine tetrahydrochloride (DAB) in 0.05 mol/L Tris-HCl buffer (pH 7.4) containing 0.01% H_2_O_2_, or a 10-minute exposure to 3,38,5,58-tetramethylbenzidine in buffer. Some sections were counterstained with hematoxylin or nuclear fast red.

To evaluate the role of annexin V, anti**-**αvβ3, and lactadherin on a time course of platelet clearance in LPS-induced sepsis, 7- to 9-week-old male C57BL/6 mice were injected with LPS (0.2 mg/kg intravenously [IV]) or sterile saline as control, and perfusion fixed at 0, 3, 6, 9, 12 and 24 h after injection. In order to evaluate the organ-specific distribution and activation of platelets, gadolinium trichloride (GdCl_3_, 20 mg/kg, IV) was administered 6 h before the LPS injection in some animals^55^. Annexin V, anti**-**αvβ3, or lactadherin was injected 2 h after GdCl_3_ injection (4 h before LPS injection) and the liver of each animal was perfusion-fixed as above. Samples of the liver were taken for microscopic examinations. Paraffin sections (4 µm) stained using immunohistochemistry as well as semi-thin sections (0.2 µm) stained with toluidine blue were analyzed by light microscopy. To quantify the platelet number, we used our previous method^30^ and the cell count is presented as cell number/mm^2^ and each value was expressed as mean ± SD of 10 mice. This study conformed to the guidelines for the care and use of laboratory animals established by the Animal Care Committee of Harvard Medical School.

### Clotting times of platelets

Platelets (2 × 10^6^) with or without opsonization by 2 nM lactadherin were co-cultured with ECs (1 × 10^4^) in 12-well culture plates for 1 h. The mixed cells were then harvested and resuspended in 100 µl Tyrode’s buffer. Platelets (2 × 10^6^) that were not cultured with ECs were suspended in the same amount of Tyrode’s and used as controls. Clotting time was determined by a one-stage recalcification time assay in a STart4-coagulometer (Diagnostica Stago)^23^. Briefly, 100 µl cell suspension was incubated with 100 µl platelet-poor plasma. After incubation for 180 seconds at 37 °C, 100 µl of warmed CaCl_2_ (1.5 mM, final) was added to start the reaction and the clotting time was recorded immediately. Tail bleeding time was measured as previously described with some modification^56^. Briefly, tail bleeding time was determined 30 min post challenge by removing 1 mm of the distal mouse tail and immersing the tail in 37 °C PBS. A complete cessation of bleeding for >30 sec was defined as the bleeding time.

### Intrinsic FXa, extrinsic FXa and prothrombinase assays

The formation of intrinsic FXa, extrinsic FXa, and prothrombinase was detected with the method described previously with some modifications^27^. To evaluate the production of intrinsic FXa, cells were incubated with 1 nM factor IXa, 5 nM factor VIII, 0.2 nM thrombin, 130 nM factor X, and 1.5 mM CaCl_2_ in factor Xa buffer (TBS with 0.2% BSA) at RT for 5 min. The reaction was then stopped by the addition of EDTA to 7 mM final concentration. After the addition of 10 μl S-2765 (0.8 mM, final), generation of factor Xa was quantified immediately at 405 nm on an automatic microplate reader (Tecan Infinite M200) in kinetic mode. The activation of extrinsic FXa was performed with 130 nM factor X, 1 nM factor VIIa, and 1.5 mM CaCl_2_ added to the samples. Measurement of extrinsic FXa was similar to that of intrinsic FXa. Results were evaluated against the rate of substrate cleavage of a standard dilution of FXa.

To measure thrombin generation, cells were incubated with 1 nM factor Va, 0.05 nM factor Xa, 1 μM prothrombin, and 1.5 mM CaCl_2_ in prothrombinase buffer (TBS with 0.05% BSA) for 5 min at RT. Thrombin production was measured with 10 μl S-2238 (0.8 mM, final) after the addition of EDTA at 405 nm in the kinetic microplate reader. Fibrin clots were evaluated as previously described^57^. Briefly, isolated platelets or a mixture of platelets and ECs were added to re-calcified (1.5 mM, final) pooled platelet-free plasma (86.7% plasma, final) in the absence or presence of 2 nM lactadherin. Peak turbidities (A_405_) of fibrin clots were quantified using the Tecan microplate reader.

### Statistical analysis

Results are presented as mean ± standard deviation (SD) of at least triplicate measurements. Statistical analysis was performed with Student t-test or ANOVA as appropriate. P < 0.05 was considered statistically significant.

## Electronic supplementary material

Supplemental information
