# Publisher Correction: Phosphatidylserine-mediated platelet clearance by endothelium decreases platelet aggregates and procoagulant activity in sepsis

**DOI:** 10.1038/s41598-018-24187-4

**Published:** 2018-04-13

**Authors:** Ruishuang Ma, Rui Xie, Chengyuan Yu, Yu Si, Xiaoming Wu, Lu Zhao, Zhipeng Yao, Shaohong Fang, He Chen, Valerie Novakovic, Chunyan Gao, Junjie Kou, Yayan Bi, Hemant S. Thatte, Bo Yu, Shufen Yang, Jin Zhou, Jialan Shi

**Affiliations:** 1 Department of Hematology of the First Hospital, Harbin Medical University, Harbin, China; 2 The Key Laboratory of Myocardial Ischemia, Ministry of Education, Heilongjiang Province, Harbin Medical University, Harbin, China; 3 Department of Medicine of the Third Hospital, Harbin Medical University, Harbin, China; 4 Department of Cardiology of the Second Hospital, Harbin Medical University, Harbin, China; 5 Departments of Cardiology of the First Hospital, Harbin Medical University, Harbin, China; 6 Department of Pathology, Harbin Medical University, Harbin, China; 7 Departments of Research VA Boston Healthcare System, Harvard Medical School, Boston, Massachusetts USA; 8 Departments of Surgery, Brigham and Women’s Hospital, VA Boston Healthcare System, Harvard Medical School, Boston, Massachusetts USA

Correction to: *Scientific Reports* 10.1038/s41598-017-04773-8, published online 10 July 2017

The original PDF version of this Article contained an error in the order of corresponding authors.

Correspondence and requests for materials should be addressed to S.Y. (email: shufen_yang@126.com) or J.Z. (email: jin_zhouxy@163.com) or J.S. (email: jialan_shi@hms.harvard.edu)

now reads:

Correspondence and requests for materials should be addressed to J.S. (email: jialan_shi@hms.harvard.edu) or J.Z. (email: jin_zhouxy@163.com) or S.Y. (email: shufen_yang@126.com)

This has now been corrected in the PDF version of this Article.

