# Supplementary figures and images for: Phosphatidylserine-mediated platelet clearance by endothelium decreases platelet aggregates and procoagulant activity in sepsis
Source: Sci Rep. 2017 Jul 10;7:4978. doi: 10.1038/s41598-017-04773-8 (PMC5504060; doi:10.1038/s41598-017-04773-8)

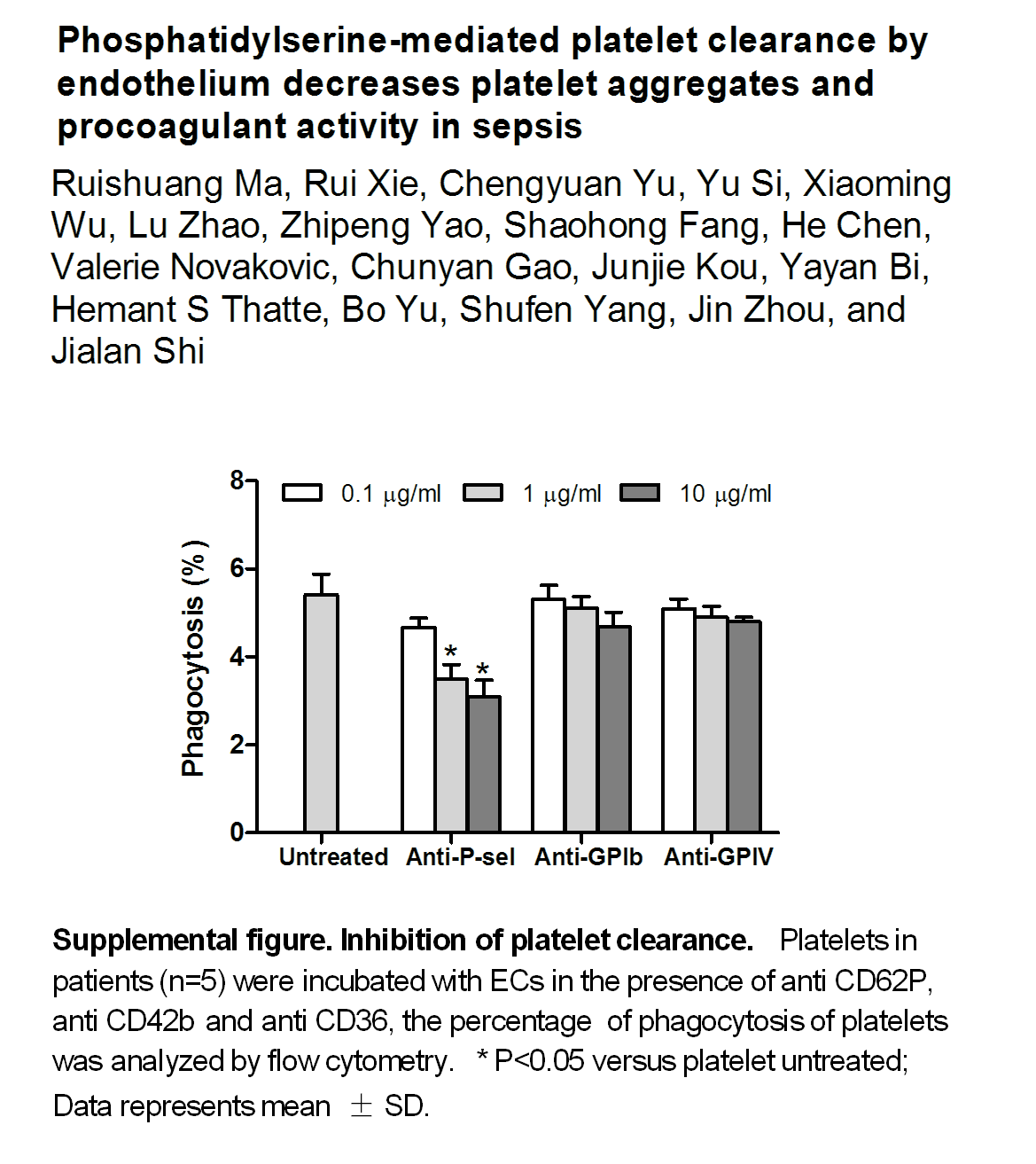

Supplement: Supplementary file 1 — Supplemental information [file 41598_2017_4773_MOESM1_ESM.tif]
